# A mathematical model for COVID-19 considering waning immunity, vaccination and control measures

**DOI:** 10.1038/s41598-023-30800-y

**Published:** 2023-03-03

**Authors:** Subhas Kumar Ghosh, Sachchit Ghosh

**Affiliations:** 1 National Australia Bank, Sydney, NSW 2000 Australia; 2 The University of Sydney, Camperdown, NSW 2006 Australia

**Keywords:** Applied mathematics, Viral infection, Scientific data

## Abstract

In this work we define a modified SEIR model that accounts for the spread of infection during the latent period, infections from asymptomatic or pauci-symptomatic infected individuals, potential loss of acquired immunity, people’s increasing awareness of social distancing and the use of vaccination as well as non-pharmaceutical interventions like social confinement. We estimate model parameters in three different scenarios—in Italy, where there is a growing number of cases and re-emergence of the epidemic, in India, where there are significant number of cases post confinement period and in Victoria, Australia where a re-emergence has been controlled with severe social confinement program. Our result shows the benefit of long term confinement of 50% or above population and extensive testing. With respect to loss of acquired immunity, our model suggests higher impact for Italy. We also show that a reasonably effective vaccine with mass vaccination program are successful measures in significantly controlling the size of infected population. We show that for a country like India, a reduction in contact rate by 50% compared to a reduction of 10% reduces death from 0.0268 to 0.0141% of population. Similarly, for a country like Italy we show that reducing contact rate by half can reduce a potential peak infection of 15% population to less than 1.5% of population, and potential deaths from 0.48 to 0.04%. With respect to vaccination, we show that even a 75% efficient vaccine administered to 50% population can reduce the peak number of infected population by nearly 50% in Italy. Similarly, for India, a 0.056% of population would die without vaccination, while 93.75% efficient vaccine given to 30% population would bring this down to 0.036% of population, and 93.75% efficient vaccine given to 70% population would bring this down to 0.034%.

## Introduction

In December 2019, an outbreak occurred in Wuhan, China involving a zoonotic coronavirus, similar to the SARS coronavirus and MERS coronavirus^1^. Subsequently, the virus has been named Severe Acute Respiratory Syndrome Coronavirus 2 (SARS-CoV-2), and the disease caused by the virus has been named the coronavirus disease 2019 (COVID-19). Since then the ongoing pandemic has seen emergence and spread of the Alpha B.1.1.7, Beta B.1.351, Gamma P.1, Delta B.1.617.2, and Omicron B.1.1.529, BA.1, BA.1.1, BA.2, BA.3, BA.4 and BA.5 VOCs as waves of infection with varied infectivity and so far the pandemic has infected more than 600 million people and has caused more than 6 million deaths worldwide.

Patients with SARS-CoV-2 infections have mild to severe respiratory illness with symptoms such as fever, cough and shortness of breath. For majority of the patients, these symptoms appear 2–14 days after exposure, and for majority the symptoms are not life threatening. However, it has been reported that there are patients who are diagnosed by a positive RT-PCR test but are either asymptomatic or minimally symptomatic^2–6^. There are reasonable evidence to consider that such asymptomatic or minimally symptomatic individuals have longer duration of viral shedding than the symptomatic individuals and can spread the virus to susceptible group^6^. Hence, it is possible that the spread of SARS-CoV-2 is much higher and such undetermined transmission has been playing an important role in sustaining the community spread.

Mathematical modelling plays an important role in understanding the trajectory of epidemic and design effective control measures under set of assumptions^7–9^. Here, we propose a new deterministic compartmental model for the COVID-19 epidemic that extends the classical SEIR (susceptible, exposed, infectious, recovered) model. We define the model and its parameters to address three different scenarios—in Italy, where there is a growing number of cases and re-emergence of the epidemic, in India, where there are significant number of cases post confinement period and in Victoria, Australia where a re-emergence has been controlled with severe social confinement program. In our model we also consider long term scenarios including re-emergence, re-infection, and control measures like mass vaccination program. Other than simulating these scenarios, another important distinction of our model is that we use parametric functions for contact rate, testing and vaccination.

Compartmental models that involve non-linear dynamical systems based on some combination of susceptible/S, infected/I, recovered/R together with some additional compartments have been utilized to predict the initial evolution of this pandemic^9–12,13^. Earlier models also include questions on effectiveness of using masks in public^14^. With the progress on efficacious vaccines being developed by late 2020, these models needed to include the vaccination rates, its effectiveness and possibility of waning immunity acquired from infection or vaccination^15^. In an earlier version of this work^16^ we have considered vaccination rates, its effectiveness and possibility of waning immunity. Waning immunity was modelled considering asymptomatic and symptomatic compartments^17^. Optimal control based strategies in minimizing number of infections over a time horizon has been considered including economic aspects^18^. Since early 2021, it became important to model and understand emergence of increasingly contagious mutations to design effective mitigation policy^19^. Non-linear coupled dynamical system is introduced in^20^. To understand probability of emergence and establishment of a vaccine-resistant strain an extended SIR model with initial stochastic dynamics was presented^21^, where every individual infected with a strain there is a small probability that a vaccine-resistant strain emerges in that individual. An alternative model using mutation probability of nucleotides was presented in^22^. A model with continuous emergence of new strains with a rate that is dependent on number of infected individuals was presented^23^. Emergence and spread of the Alpha B.1.1.7, Delta B.1.617.2, and Omicron B.1.1.529 VOCs including cross infection across age restricted compartmental model was presented in^24^.

**Figure 1:**
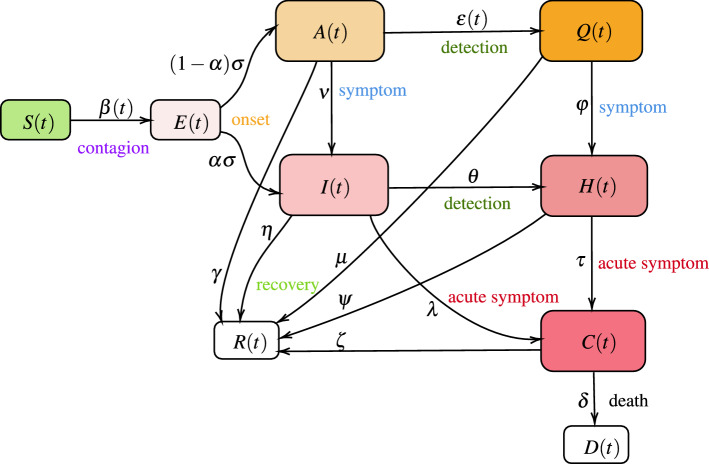
The model consists of following compartments: susceptible *S*(*t*),, exposed *E*(*t*), asymptomatic *A*(*t*), symptomatic *I*(*t*), quarantined *Q*(*t*), isolated *H*(*t*), deceased (*D*(*t*) and recovered *R*(*t*) individuals in a population of $N(t) = S(t) + E(t) + A(t) + I(t) + Q(t) + H(t) + R(t) + D(t)$ individuals.

In this work, we use a deterministic compartmental model that is an extension of the SEIR model^8,25^ in which we include current experience with SARS-CoV-2. We partition the total population into susceptible individuals (*S*(*t*)), exposed individuals (*E*(*t*)), Asymptomatic, undetected and infected individuals (*A*(*t*)), Symptomatic, undetected, and infected individuals (*I*(*t*)), Asymptomatic, diagnosed and infected individuals (*Q*(*t*)), Symptomatic, diagnosed, and infected individuals (*H*(*t*)), individuals with acute symptoms and in critical care (*C*(*t*)), and recovered (*R*(*t*)) and deceased (*D*(*t*)), see Fig. 1.

The transmission dynamics of COVID-19 in the basic model is given by the following deterministic system of non-linear differential Eqs. (1)–(10):

1 $$\begin{aligned} \frac{dE}{dt}= & {} \beta (t) \left( I + \kappa A + \omega Q + \rho H \right) \frac{S}{N} - \sigma E, \end{aligned}$$

2 $$\begin{aligned} \frac{dI}{dt}= & {} \alpha \sigma E + \nu A - \left( \eta + \theta + \lambda \right) I, \end{aligned}$$

3 $$\begin{aligned} \frac{dA}{dt}= & {} \left( 1-\alpha \right) \sigma E - \left( \varepsilon (t) + \nu + \gamma \right) A, \end{aligned}$$

4 $$\begin{aligned} \frac{dQ}{dt}= & {} \varepsilon (t) A - \left( \varphi + \mu \right) Q, \end{aligned}$$

5 $$\begin{aligned} \frac{dH}{dt}= & {} \theta I + \varphi Q - \left( \tau + \psi \right) H, \end{aligned}$$

6 $$\begin{aligned} \frac{dC}{dt}= & {} \tau H + \lambda I - \left( \delta + \zeta \right) C, \end{aligned}$$

7 $$\begin{aligned} \frac{dD}{dt}= & {} \delta C, \end{aligned}$$

8 $$\begin{aligned} \frac{dR}{dt}= & {} \left( \eta I + \gamma A + \mu Q + \psi H + \zeta C \right) , \end{aligned}$$

9 $$\begin{aligned} \frac{dS}{dt}= & {} -\beta (t) \left( I + \kappa A + \omega Q + \rho H \right) \frac{S}{N}, \end{aligned}$$

where,

10 $$\begin{aligned} N(t) = S(t) + E(t) + A(t) + I(t) + Q(t) + H(t) + R(t) + D(t), \end{aligned}$$

is the total population.

### Susceptible individuals: *S*(*t*)

In our model, the susceptible individuals gets exposed to infection, and move to exposed group *E*(*t*), from coming in contact with an infected individual, who may be symptomatic, asymptomatic, quarantined, or isolated. $\beta (t)$ is the baseline infectious contact rate, which can vary with time or assumed constant for the analysis of our baseline model. We assume that a person who is infected with symptom, and is not isolated, has the basic transmission coefficient of $\beta (t)$, that is changing over time. Based on^14,26^, we define $\beta (t)$ to have a value $\beta _0$ till time $t_0$ and then as a decreasing function with respect to time *t*, to reaching $\beta _{\min }$.

11 $$\begin{aligned} \beta (t) = {\left\{ \begin{array}{ll} \beta _0 &{} t < t_0 \\ \beta _{\min } + \left( \beta _0 - \beta _{\min }\right) e^{-r\left( t - t_0\right) } &{} t \ge t_0 \end{array}\right. } \end{aligned}$$

We would like to note that in certain countries, stringency measures were in place at an earlier stages of the epidemic and was relaxed over time leading to a higher contact rate. Under such scenario, we use an increasing function for $\beta (t)$ after an initial phase of reaching or nearing $\beta _{\min }$ as

12 $$\begin{aligned} \beta (t) = {\left\{ \begin{array}{ll} \beta _0 &{} t< t_0 \\ \beta _{\min } + \left( \beta _0 - \beta _{\min }\right) e^{-r\left( t - t_0\right) } &{} t_0 \le t < t_1\\ \beta _{new} - (\beta _{new} - \beta _{min}) e^{-u \left( t - t_1\right) } &{} t \ge t_1 \end{array}\right. } \end{aligned}$$

We assume that the asymptomatic individuals infect with a lower contact rate ($\kappa < 1$) than the symptomatic individuals. Once someone symptomatic is diagnosed, they can only infect healthcare workers and this lower contact rate is captured by the parameter ($\rho < 1$). Similarly, quarantined individuals have much lower contact rate of ($\omega < 1$). Overall rate of change for the susceptible population is thus defined by Eq. (9).

### Exposed individuals: *E*(*t*)

Individuals in compartment *E*, are exposed to the virus, and are not contagious during a period of latent time. An individual in *E* becomes infectious, and moves to compartment *A* as asymptomatic or to *I* as symptomatic. We assume that $\sigma$ is the transition rate from exposed to infectious, and a fraction $\alpha$ of them show symptoms. It is important to note that following ideas from other extended SEIR model e.g.^14,17,27–29^ we have extended standard SEIR model to include symptomatic and asymptomatic classes, and the fact the in SARS-CoV-2, it has been observed that asymptomatic classes can infect susceptible individuals^28^. Overall rate of change for the exposed population is thus defined by Eq. (1).

### Symptomatic individuals: *I*(*t*)

Symptomatic individuals can get diagnosed ($\theta$) and be isolated, or show acute symptoms and be hospitalized ($\lambda$), or can recover at the rate $\eta$. It has been observed that $\eta \ge \gamma$, where symptomatic individuals recover at faster rate than asymptomatic individuals, and asymptomatic individuals have longer duration of viral shedding^6^. Overall rate of change is given by Eq. (2).

### Asymptomatic individuals: *A*(*t*)

Asymptomatic individuals can eventually show symptoms and move to *I* at rate $\nu$ or can have a positive diagnosis and move to quarantine. We model testing of asymptomatic population as a function of time as the community testing process ramps up. Testing rate has been captured as $\varepsilon (t)$. Finally, they can recover at the rate $\gamma$. Overall rate of change is given by Eq. (3). A note on testing rate is in order. We model it as following:

13 $$\begin{aligned} \varepsilon (t) = {\left\{ \begin{array}{ll} \varepsilon _0 &{} t < t_2 \\ \varepsilon _{\max } - (\varepsilon _{\max } - \varepsilon _0) e^{-s \left( t-t_2\right) } &{} t > t_2 \end{array}\right. } \end{aligned}$$

Here, we set the testing rate as an increasing function of time, because of the increasing production of detection kits and the improvement of detection techniques. Where, $1/ \varepsilon _0$ can be thought of waiting time for an individual to get tested in the start of an outbreak and $1/\varepsilon _{\max }$ is the reduced waiting time at a later point during the epidemic. In other words, $\lim _{t \rightarrow \infty } \varepsilon (t) = \varepsilon _{\max } \ge \varepsilon _0$.

### Quarantined individuals: *Q*(*t*)

Quarantined individuals are asymptomatic population after diagnosis and they have very low contact rate ($\omega < 1$). This is mostly by infecting the other family members or by breach of protocol. They however, may develop symptoms and move to *H* or recover. Overall rate of change is given by Eq. (4).

### Isolated individuals: *H*(*t*)

Isolated individuals are showing symptoms and has been either home isolated or has been hospitalized. They can pass the infection to a limited number of health care professional or caregiver ($\rho$). They can become critical and require treatments in intensive care ($\tau$), and a large number of them recover ($\psi$). Overall rate of change is given by Eq. (5).

### Critical, recovered and deceased individuals: *C*(*t*), *R*(*t*), *D*(*t*)

These counters collect information on population that are critical, recovered or have deceased. Overall rate of change is given by Eqs. (6)–(8). We assume that in the base model, recovered individuals possess lasting immunity against SARS-CoV-2 over the period of simulation, however, we extend the model to consider the possibility of re-infection in later section of this paper.

## Results

### Italy

For the COVID-19 epidemic in Italy, we estimate the model parameters based on data from 24th February 2020 (day 1) to 5th March 2022 (day 740) and show the effects of social confinement that is in effect since 9 March 2020, in controlling the spread of the epidemic, and subsequently easing the restriction from 4th May 2020 has again increased contact rate leading to re-emergence. We also model possible longer-term scenarios illustrating the effects of different countermeasures, including social distancing, population-wide testing, vaccination to contain SARS-CoV-2, as well as possible effects of re-infection and loss of immunity.

**Figure 2:**
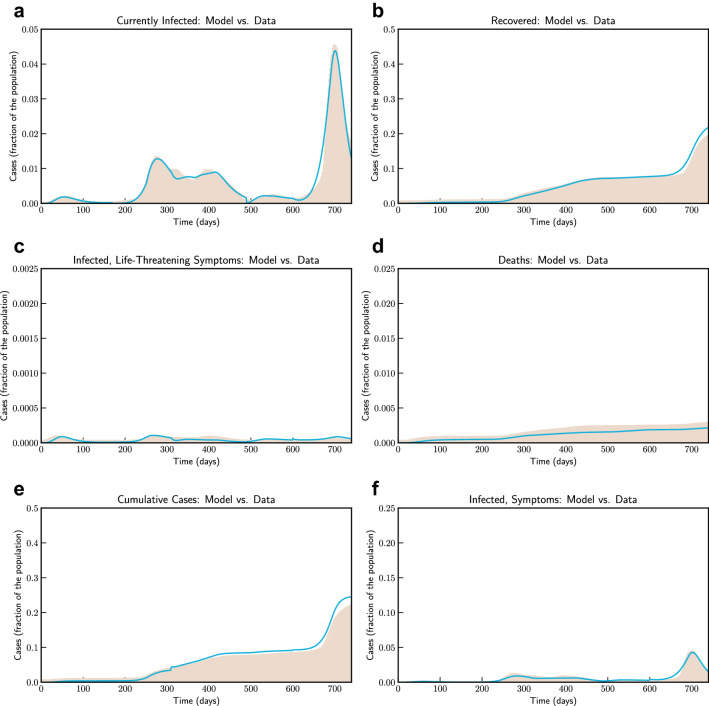
Model simulation compared to real data (Italy)—Comparison between the official data (histogram) and the results with our model. Description of panels: (**a**) Number of currently active cases, $\left( Q(t) + H(t) + C(t)\right)$, (**b**) number of reported recovered individuals. $\int _{0}^{t}{\left( \mu Q(s) + \psi H(s) + \zeta C(s) \right) ds}$, (**c**) number of reported infected with life-threatening symptoms, admitted to ICU, *C*(*t*), (**d**) Number of deceased individuals *D*(*t*), (**e**) number of reported infected with no (or mild) symptoms, who are quarantined at home. *Q*(*t*) , (**f**) number of reported infected with symptoms, who are hospitalized. *H*(*t*).

Model simulation compared to real data is shown in Fig. 2. We have estimated $R_0$ according to Eq. (eqn:Rnot). In our estimate $R_0 = 4.09$ in the beginning and then reduces to 0.779 after 45 days, and to 0.18 after 90 days. Subsequently, after 110 days it starts increasing and reaches a value 1.67 on day 186.

**Figure 3:**
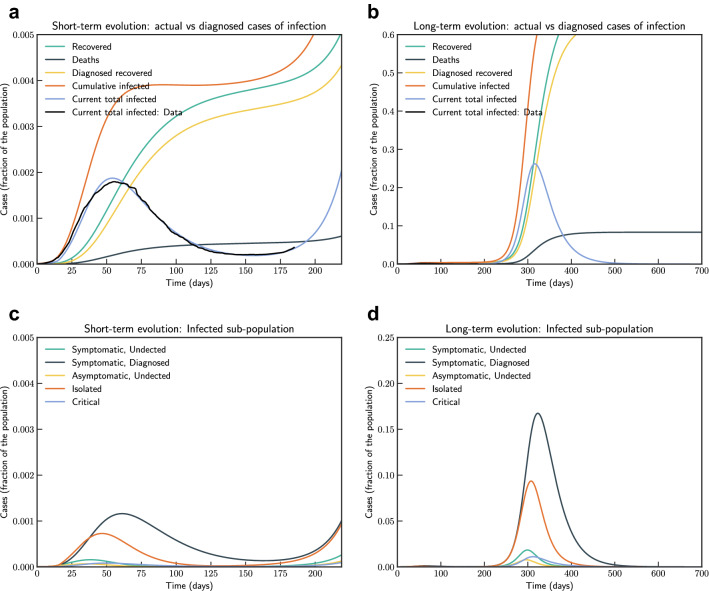
Model simulation compared to real data (Italy)—Epidemic evolution predicted by the model based on the available data. Description of panels: (**a**,**c**) The short-term epidemic evolution obtained by reproducing the data trend with the model, (**b**,**d**) Long term epidemic evolution over 700 days. Plots refers to all cases of infection, both diagnosed and non-diagnosed, predicted by the model, although non-diagnosed cases are of course not counted in the data. Note that not all panels are in the same scale.

**Figure 4:**
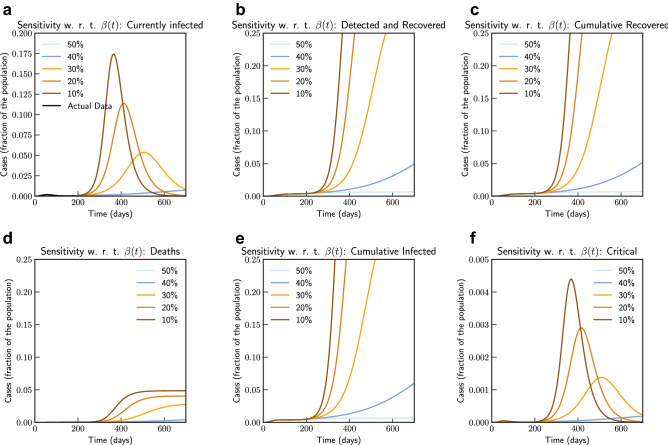
Sensitivity of $\beta (t)$ with $50\%, 40\%, 30\%, 20\%$ and $10\%$ reduction from current contact rate (Italy). Note that not all panels are in the same scale.

In Fig. 3, we show both short term and long term evolution after fitting the model to data. Result shows that without any control measure in long term a significant portion of population getting infected. Hence, we consider the effect of continued social distancing, awareness and confinement measures by sensitivity analysis of contact rate parameter $\beta (t)$. We consider reducing $\beta (t)$ by $50\%, 40\%, 30\%, 20\%$ and $10\%$ from current contact rate. Figure 4, indicates that reducing contact rate by half can reduce a potential peak infection of 15% population to less than 1.5% of population, and potential deaths from 0.48 to 0.04%.

**Figure 5:**
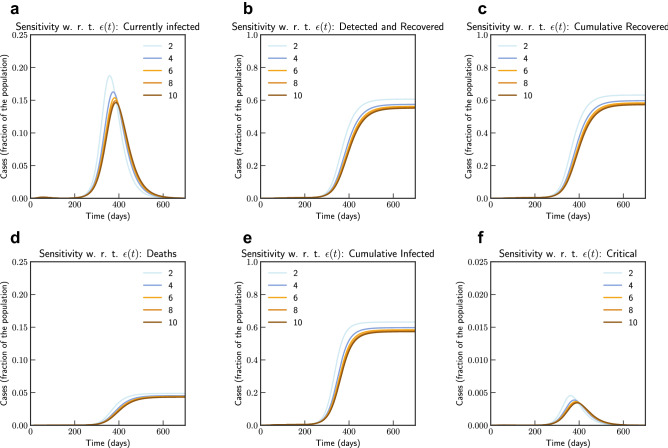
Sensitivity of $\varepsilon (t)$ with changed rate of testing by 2, 4, 6, 8 and 10 times the current rate (Italy). Note that not all panels are in the same scale.

Subsequently, we also consider the effects of testing and rate of detection of latent cases $\varepsilon (t)$. We consider changing the current testing rate by 2, 4, 6, 8 and 10 times the current rate. Figure 5, the simulation of the model after fitting current data indicates that an increased testing rate by 10 times will reduce potential peak infection rate from 18% of population to 14%, deaths from 0.48 to 0.42%. Our model confirms that extensive testing campaigns can reduce the infection peak (as the diagnosed population enters quarantine and is therefore less likely to affect the susceptible population) and help end the epidemic more quickly. However, it can be observed that sensitivity of contact rate is much more significant than testing. This is also due to that fact that in our model we have considered $\varepsilon$ as a parameter that impacts how asymptomatic cases are moved to quarantine, while infection from asymptomatic cases are lower compared to symptomatic cases.

### India

India had one of the most strict stay home order across the country in first phase of the lock-down between 25 March 2020 and 14 April 2020 (21 days), where an entire population of 1.3 billion people was put under restricted movement. Overall the lock-down had multiple phases, second phase was from 15th of April 2020 to 3rd of May 2020, and third phase was 4th of May to 17th of May, 2020.

**Figure 6:**
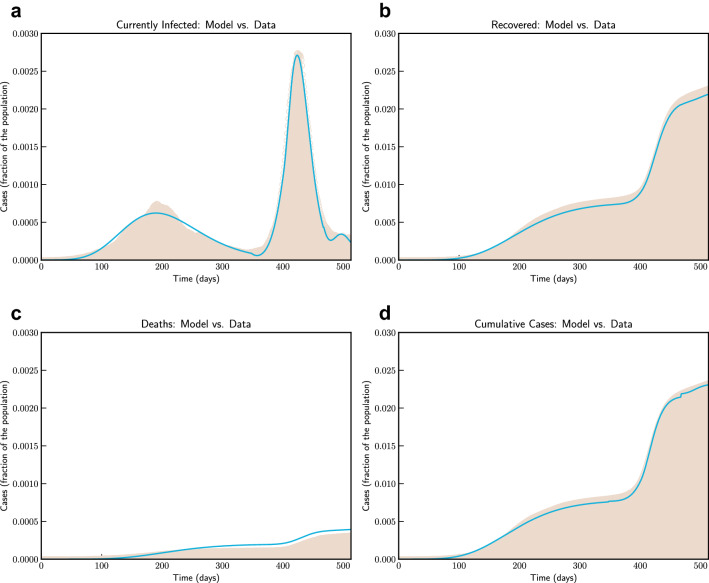
Model simulation compared to real data (India)—Comparison between the official data (histogram) and the results with our model. Description of panels: (**a**) Number of currently active cases, $\left( Q(t) + H(t) + C(t)\right)$, (**b**) number of reported recovered individuals. $\int _{0}^{t}{\left( \mu Q(s) + \psi H(s) + \zeta C(s) \right) ds}$, (**c**) Number of deceased individuals *D*(*t*).

We use data from Johns Hopkins University Center for Systems Science and Engineering (JHU CSSE). We use data from 9nd March 2020 (Day 1) to 4th August 2021 (Day 514). Figure 6 compares the fitted model with actual data. From August 2021 onwards recovery data is not available and hence we could not compare the model with actual number of currently active cases to which we fit our model for parameter estimation. Based on our estimate, initial value of $R_0$ was 1.762, which reduces to 1.68 after a month when lockdown as in place. At the end of lockdown phases it is about 1.5, and finally after 194 days it reaches 1.03. These values are comparable with findings in^30,31^.

**Figure 7:**
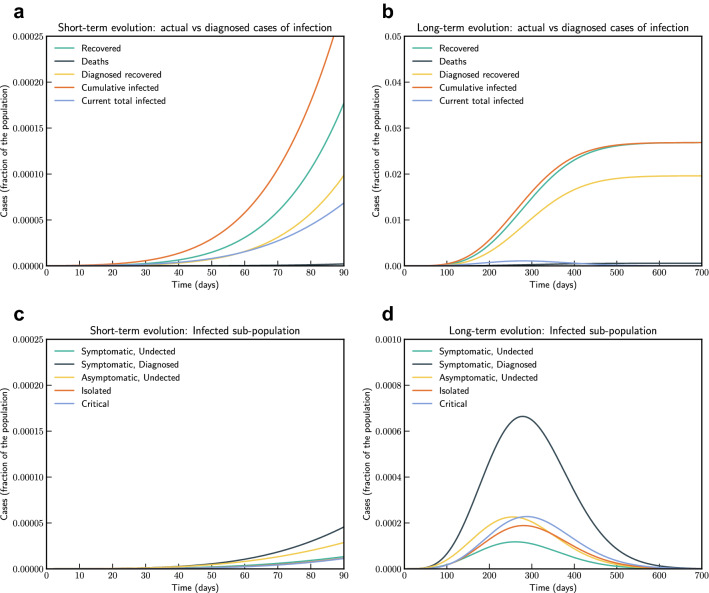
Model simulation compared to real data (India)—Epidemic evolution predicted by the model based on the available data. Description of panels: (**a**,**c**) The short-term epidemic evolution obtained by reproducing the data trend with the model for 90 days, (**b**,**d**) Long term epidemic evolution over 700 days. Plots refers to all cases of infection, both diagnosed and non-diagnosed, predicted by the model, although non-diagnosed cases are of course not counted in the data. Note that not all panels are in the same scale.

We have simulated the long term evolution of the epidemic for India after estimating the parameters as shown in Fig. 7. Simulation suggested that peak in India for the first wave reached around 275 days from 2nd March 2020 with about 2.6% population getting infected.

**Figure 8:**
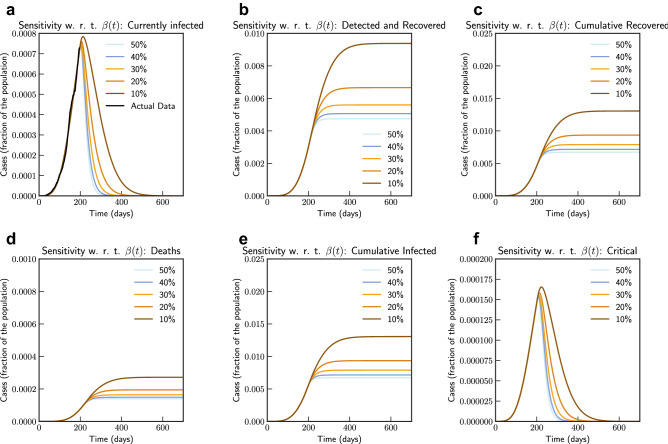
Sensitivity of $\beta (t)$ with $50\%, 40\%, 30\%, 20\%$ and $10\%$ reduction from current contact rate (India). Note that not all panels are in the same scale.

**Figure 9:**
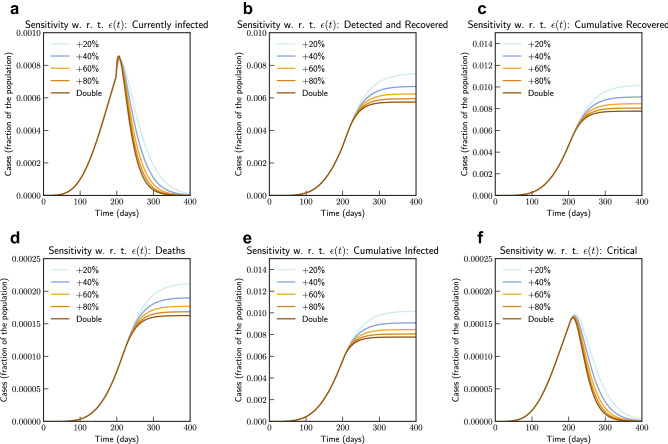
Sensitivity of $\varepsilon (t)$ with changed rate of testing by 1.2, 1.4, 1.6, 1.8 and 2 times the current rate (India). Note that not all panels are in the same scale.

Simulation studies on impact of reducing contact rate and testing is captured in Figs. 8 and 9 respectively. From the simulation we can observe that the sensitivity to contact rate is higher compared to increased testing. A reduction in contact rate by 50% compared to a reduction of 10% can reduce death from 0.0268% to 0.0141% of population.

### Victoria, Australia

**Figure 10:**
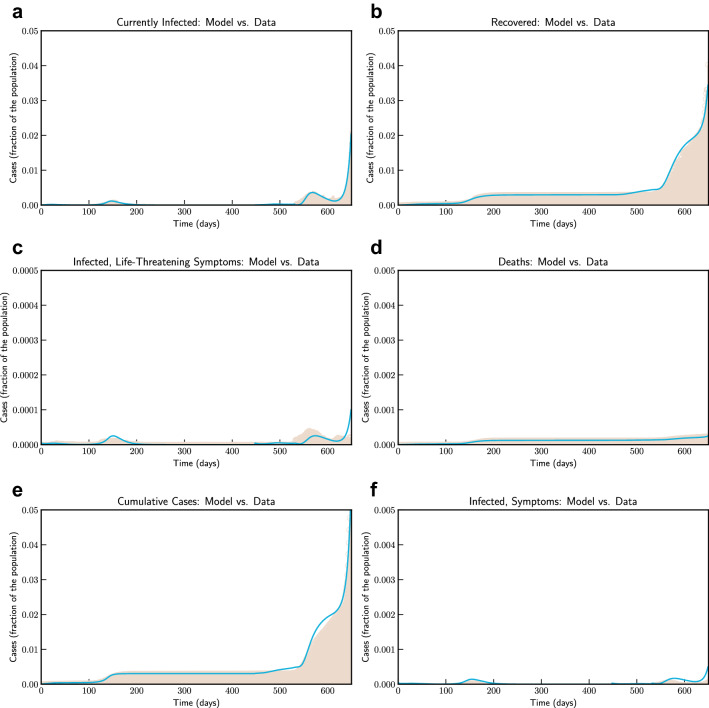
Model simulation compared to real data (Victoria, Australia)–Comparison between the official data (histogram) and the results with our model. Description of panels: (**a**) Number of currently active cases, $\left( Q(t) + H(t) + C(t)\right)$, (**b**) number of reported recovered individuals. $\int _{0}^{t}{\left( \mu Q(s) + \psi H(s) + \zeta C(s) \right) ds}$, **(c):** number of reported infected with life-threatening symptoms, admitted to ICU, *C*(*t*), (**d**) Number of deceased individuals *D*(*t*), (**e**) Cumulative number of cases, $Q(t) + H(t) + C(t) + D(t) + \int _{0}^{t}{\left( \mu Q(s) + \psi H(s) + \zeta C(s) \right) ds}$ (**f**) number of reported infected with symptoms, who are hospitalized. *H*(*t*).

**Figure 11:**
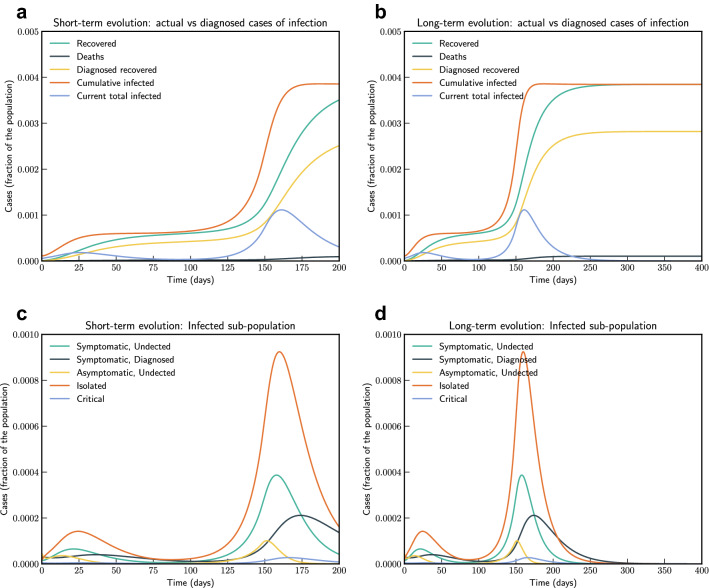
Model simulation compared to real data (Victoria, Australia)—Epidemic evolution predicted by the model based on the available data. Description of panels: (**a**,**c**) The short-term epidemic evolution obtained by reproducing the data trend with the model, (**b**,**d**) Long term epidemic evolution over 350 days. Plots refers to all cases of infection, both diagnosed and non-diagnosed, predicted by the model, although non-diagnosed cases are of course not counted in the data. Note that not all panels are in the same scale.

For Victoria, Australia we use data from 11th March, 2020 (day 1) to 13th January, 2022 (day 650). Figure 10 compares the fitted model with actual data. Consistent with other places Victoria had initial outbreak during March and April 2020—which was effectively controlled. Control measures were relaxed during June 2020 to revive economic activities. Based on our estimate $R_0$ in Victoria was 2.72 in the beginning of July. A stricter confinement was placed in selected localities on 1 July 2020, which was extended to the whole of metropolitan Melbourne and Mitchell Shire on 8 July. We have simulated the long term evolution of the epidemic for Victoria, after estimating the parameters as shown in Fig. 11. Estimated by our model, $R_0$ was 2.86 at that time. However, the restriction was effective to bring down $R_0$ to 1.13 by 30, July, 2020. We note that our findings are similar to^32^.

## Methods

### Baseline mathematical models

#### Baseline epidemiological parameters

In this section we describe the estimated values of various parameters based on the current literature. It has been noted in the literature that the clinical course of the disease is typically quite long. Average total duration of illness has been estimated to be three weeks in^33^. Parameter $\beta _0$ is strongly dependent on the population behaviour. We select a default value that has been estimated in^34^ for pre-lockdown period. Baseline values for various parameters are listed in the Table 1.

**Table 1:** Baseline parameters, brief description, possible ranges based on modeling and clinical studies, and default value chosen for this study. Note that these parameters were estimated to fit the model to real data.

Param	Description	Possible range	Default
$\beta$	infectious contact rate	0.5–1.5 $\text{day}^{-1}$^34,35^	1.14 $\text{day}^{-1}$
$\kappa$	infectiousness factor asymptomatic	0.4–0.6^35,36^	0.5
$\omega$	infectiousness factor quarantined	0.005–0.0114^9^	0.0114
$\rho$	infectiousness factor isolated	0.005–0.0114^9^	0.0114
$\sigma$	transition rate exposed to infectious	1/14–1/3 $\text{day}^{-1}$^35,37^	1/5.2 $\text{day}^{-1}$
$\alpha$	fraction of infections that become symptomatic	0.15–0.7^35,36,38^	0.3
$\nu$	transition rate asymptomatic to symptomatic	0.025–0.125^9^	0.125
$\varepsilon$	detection rate asymptomatic	0.171^9^	0.171
$\varphi$	rate of quarantined to isolation	0.025–0.125^9^	0.125
$\theta$	rate of detection of symptomatic	0.371^9^	0.371
$\tau$	rate of developing life-threatening symptoms in isolation	0.027^9^	0.027
$\lambda$	rate of developing life-threatening symptoms for symptomatic	0.017^9^	0.017
$\gamma$	recovery rate of asymptomatic	0.034^9^	0.034
$\eta$	recovery rate of symptomatic	0.017^9^	0.017
$\mu$	recovery rate of quarantined	0.034^9^	0.034
$\psi$	recovery rate of isolated	0.017^9^	0.017
$\zeta$	recovery rate of critical	0.017^9,38^	0.017
$\delta$	mortality rate	0.01–0.05^9,36,39^	0.01

#### The basic reproduction number for baseline model

The basic reproduction number is calculated for the special case when we have $\beta (t) = \beta _0, \varepsilon (t) = \varepsilon _0$. In following we explore the local stability of the disease-free equilibrium (DFE) using the next generation operator method^40,41^. Following^41^, we define the system of Eqs. (1)–(10), in more compact form as:

14 $$\begin{aligned} \dot{X} = f(X) = \mathscr {F}(X) - \mathscr {V}(X), \end{aligned}$$

where, $X = \left( E, I, A, Q, H, C, D, R, S \right) ^t$, and $\mathscr {F}(X)$ containing rate of appearance of new infections defined as:

15 $$\begin{aligned} \mathscr {F}(X) = \begin{pmatrix} \beta _0 \left( I + \kappa A + \omega Q + \rho H \right) \frac{S}{N} \\ 0\\ 0\\ 0\\ 0\\ 0\\ 0\\ 0\\ 0 \end{pmatrix}, \end{aligned}$$

and, $\mathscr {V}(X)$ capturing the movement between the compartments, with $\mathscr {V}^{-}(X)$ as the rate of outward transfer, and $\mathscr {V}^{+}(X)$ as the rate of inward transfer for each compartment, we have,

16 $$\begin{aligned} \begin{aligned} \mathscr {V}(X)&= \mathscr {V}^{-}(X) - \mathscr {V}^{+}(X)\\&= \begin{pmatrix} \sigma E \\ \left( \eta + \theta + \lambda \right) I\\ \left( \varepsilon _0 + \nu + \gamma \right) A\\ \left( \varphi + \mu \right) Q\\ \left( \tau + \psi \right) H\\ \left( \delta + \zeta \right) C\\ 0\\ 0\\ \beta _0 \left( I + \kappa A + \omega Q + \rho H \right) \frac{S}{N} \end{pmatrix} - \begin{pmatrix} 0 \\ \alpha \sigma E + \nu A\\ \left( 1 - \alpha \right) \sigma E \\ \varepsilon _0 A\\ \theta I + \varphi Q\\ \tau H + \lambda I\\ \delta C\\ \left( \eta I + \gamma A + \mu Q + \psi H + \zeta C \right) \\ 0 \end{pmatrix}\\ \mathscr {V}(X)&= \begin{pmatrix} \sigma E \\ \left( \eta + \theta + \lambda \right) I - \alpha \sigma E - \nu A\\ \left( \varepsilon _0 + \nu + \gamma \right) A - \left( 1 - \alpha \right) \sigma E\\ \left( \varphi + \mu \right) Q - \varepsilon _0 A\\ \left( \tau + \psi \right) H - \theta I - \varphi Q\\ \left( \delta + \zeta \right) C - \tau H - \lambda I\\ -\delta C\\ -\left( \eta I + \gamma A + \mu Q + \psi H + \zeta C \right) \\ \beta _0 \left( I + \kappa A + \omega Q + \rho H \right) \frac{S}{N} \end{pmatrix} \end{aligned} \end{aligned}$$

We also define $\mathscr {X}_s$, as the set of all possible disease free states. In order to directly apply the results in^41^, following shall hold for equation $\dot{X} = f(x) = \mathscr {F}(X) - \mathscr {V}(X)$:

1. Functions $\mathscr {F}(X)$, $\mathscr {V}^{-}(X)$ and $\mathscr {V}^{+}(X)$, are all non-negative, when $X > 0$.
2. If $X \in \mathscr {X}_s$, then $\mathscr {V}^{-}(x)=\mathscr {F}(x)=\mathscr {V}^{+}(x)=0$ for $x \in \{E, I, A, Q, H, C, D, R\}$.
3. Let $Df(X_0)$ be the Jacobian matrix evaluated at DFE $X_0$, and defined as the partial derivative $[{\partial f}/{\partial x}]$ for $x \in \{E, I, A, Q, H, C, D, R, S\}$. If $\mathscr {F}(X) = 0$, then all eigenvalues of $Df(X_0)$ has negative real parts.

We note that each function represents a directed transfer of individuals, and they are all non-negative. (1) and (2), can be observed from the Eqs. (15) and (16). For (3), setting $\mathscr {F}(X) = 0$, we consider linearized system $\dot{X} = - D\mathscr {V}(X_0)(X-X_0)$, near DFE. From Eq. (17) we observe that eigenvalues corresponding to $Df(X_0)$ has zero eigenvalues of multiplicity 3 with associated eigenvectors in the directions of *D*, *R*, *S*. The results in^41^ still holds for our system for stability in the directions of the susceptible and recovered compartment (note that )as *D* is a counting compartment), this however, has no consequence in the meaning of the threshold $\mathscr {R}_0$. In fact this technicality can be resolved by adding natural birth and death rates proportional to the compartments *S* and *R* that is arbitrarily small and positive. Let $X_0 \in \mathscr {X}_s$ be a DFE. Then $X_0 = \left( 0, 0, 0, 0, 0, 0, 0, 0, S_0 \right)$, and with $S_0/N_0=1$ we have,

$$\begin{aligned} \begin{aligned} D \mathscr {F}(X_0) = \begin{pmatrix} 0 &{} \beta _0 &{} \kappa \beta _0 &{} \omega \beta _0 &{} \rho \beta _0 &{} 0 &{} 0 &{} 0 &{} 0 \\ 0 &{} 0 &{} 0 &{} 0 &{} 0 &{} 0 &{}0 &{} 0 &{}0 \\ 0 &{} 0 &{} 0 &{} 0 &{} 0 &{} 0 &{}0 &{} 0 &{}0 \\ 0 &{} 0 &{} 0 &{} 0 &{} 0 &{} 0 &{}0 &{} 0 &{}0 \\ 0 &{} 0 &{} 0 &{} 0 &{} 0 &{} 0 &{}0 &{} 0 &{}0 \\ 0 &{} 0 &{} 0 &{} 0 &{} 0 &{} 0 &{}0 &{} 0 &{}0 \\ 0 &{} 0 &{} 0 &{} 0 &{} 0 &{} 0 &{}0 &{} 0 &{}0 \\ 0 &{} 0 &{} 0 &{} 0 &{} 0 &{} 0 &{}0 &{} 0 &{}0 \\ 0 &{} 0 &{} 0 &{} 0 &{} 0 &{} 0 &{}0 &{} 0 &{}0 \end{pmatrix}\ = \begin{pmatrix} F &{} 0\\ 0 &{} 0 \end{pmatrix} \end{aligned} \end{aligned}$$

With,

$$\begin{aligned} F = \begin{pmatrix} 0 &{} \beta _0 &{} \kappa \beta _0 &{} \omega \beta _0 &{} \rho \beta _0 \\ 0 &{} 0 &{} 0 &{} 0 &{} 0\\ 0 &{} 0 &{} 0 &{} 0 &{} 0\\ 0 &{} 0 &{} 0 &{} 0 &{} 0\\ 0 &{} 0 &{} 0 &{} 0 &{} 0 \end{pmatrix} \end{aligned}$$

Similarly, we have

17 $$\begin{aligned} \begin{aligned} D \mathscr {V}(X_0) = \\&\begin{pmatrix} \sigma &{} 0 &{} 0 &{} 0 &{} 0 &{} 0 &{}0 &{} 0 &{}0 \\ -\alpha \sigma &{} \left( \eta + \theta + \lambda \right) &{} - \nu &{} 0 &{} 0 &{} 0 &{}0 &{} 0 &{}0 \\ -\left( 1- \alpha \right) \sigma &{} 0 &{} \left( \varepsilon _0 + \nu + \gamma \right) &{} 0 &{} 0 &{} 0 &{}0 &{} 0 &{}0 \\ 0 &{} 0 &{} - \varepsilon _0 &{} \left( \varphi + \mu \right) &{} 0 &{} 0 &{}0 &{} 0 &{}0 \\ 0 &{} -\theta &{} 0 &{} -\varphi &{} \left( \tau + \psi \right) &{} 0 &{}0 &{} 0 &{}0 \\ 0 &{} -\lambda &{} 0 &{} 0 &{} -\tau &{} \left( \delta + \zeta \right) &{}0 &{} 0 &{}0 \\ 0 &{} 0 &{} 0 &{} 0 &{} 0 &{} -\delta &{}0 &{} 0 &{}0 \\ 0 &{} -\eta &{} -\gamma &{} -\mu &{} -\psi &{} -\zeta &{}0 &{} 0 &{}0 \\ 0 &{} \beta _0 &{} \kappa \beta _0 &{} \omega \beta _0 &{} \rho \beta _0 &{} 0 &{}0 &{} 0 &{}0 \end{pmatrix} =\begin{pmatrix} V &{} 0\\ J_3 &{} J_4 \end{pmatrix} \end{aligned} \end{aligned}$$

With,

$$\begin{aligned} V = \begin{pmatrix} \sigma &{} 0 &{} 0 &{} 0 &{} 0\\ -\alpha \sigma &{} \left( \eta + \theta + \lambda \right) &{} - \nu &{} 0 &{} 0\\ -\left( 1- \alpha \right) \sigma &{} 0 &{} \left( \varepsilon _0 + \nu + \gamma \right) &{} 0 &{} 0\\ 0 &{} 0 &{} - \varepsilon _0 &{} \left( \varphi + \mu \right) &{} 0\\ 0 &{} -\theta &{} 0 &{} -\varphi &{} \left( \tau + \psi \right) \end{pmatrix} \end{aligned}$$

and,

$$\begin{aligned} V^{-1} = \begin{pmatrix} \frac{1}{\sigma } &{} 0 &{} 0 &{} 0 &{} 0\\ - \frac{\alpha \nu - \alpha r_{3} - \nu }{r_{1} r_{3}} &{} \frac{1}{r_{1}} &{} \frac{\nu }{r_{1} r_{3}} &{} 0 &{} 0\\ - \frac{\alpha }{r_{3}} + \frac{1}{r_{3}} &{} 0 &{} \frac{1}{r_{3}} &{} 0 &{} 0\\ - \frac{\alpha \varepsilon - \varepsilon }{r_{3} r_{4}} &{} 0 &{} \frac{\varepsilon }{r_{3} r_{4}} &{} \frac{1}{r_{4}} &{} 0\\ - \frac{\alpha \varepsilon r_{1} \varphi + \alpha \nu r_{4} \theta - \alpha r_{3} r_{4} \theta - \varepsilon r_{1} \varphi - \nu r_{4} \theta }{r_{1} r_{2} r_{3} r_{4}} &{} \frac{\theta }{r_{1} r_{2}} &{} \frac{\varepsilon r_{1} \varphi + \nu r_{4} \theta }{r_{1} r_{2} r_{3} r_{4}} &{} \frac{\varphi }{r_{2} r_{4}} &{} \frac{1}{r_{2}} \end{pmatrix} \end{aligned}$$

Defining $\rho (A) = \max {\{ | \lambda _1 |, \ldots , | \lambda _n |\} }$ as the spectral radius of an $n \times n$ matrix *A*, with eigenvalues $\lambda _1 \ldots \lambda _n$, and $|\dot{|}$ denoting absolute values. According to^41^, basic reproduction number $\mathscr {R}_0$ associated to the system can be computed as $\mathscr {R}_0 = \rho (FV^{-1})$. Hence, based on the discussion above, for the baseline model we have,

18 $$\begin{aligned} \begin{aligned} \mathscr {R}_0&= \beta _0 \left( \frac{\alpha }{r_1} + \frac{\nu \left( 1-\alpha \right) }{r_1 r_3}\right) + \kappa \beta _0 \left( \frac{1-\alpha }{r_3} \right) + \omega \beta _0 \left( \frac{\varepsilon _0 \left( 1-\alpha \right) }{r_3 r_4}\right) \\&+ \rho \beta _0 \left( \frac{\alpha \theta }{r_1 r_2} + \frac{\left( 1-\alpha \right) \varepsilon _0 \varphi }{r_2 r_3 r_4} + \frac{\left( 1-\alpha \right) \nu \theta }{r_1 r_2 r_3}\right) \end{aligned} \end{aligned}$$

where, $r_1 = \left( \eta + \theta + \lambda \right)$, $r_2 = \left( \tau + \psi \right)$, $r_3 = \left( \varepsilon _0 + \nu + \gamma \right)$, and $r_4 = \left( \varphi + \mu \right)$. When the epidemic is over, we will have the condition that $\bar{E}=\bar{I}=\bar{A}=\bar{Q}=\bar{H}=\bar{C}=0, \bar{D}\ge 0, \bar{R}\ge 0, \bar{S}\ge 0$, with $\bar{D} + \bar{R} + \bar{S} = 1$. That is, only the susceptible, the recovered and the deceased individuals are eventually present.

##### Proposition 1

The system of equation with susceptible population $\bar{S}$ is asymptotically stable if and only if

$$\begin{aligned} \bar{S} \le \bar{S}^* = \frac{1}{R_0} \end{aligned}$$

##### Proof

The dynamical system matrix of the linearized system near a DFE is given by:

$$\begin{aligned} M = \begin{pmatrix} -\sigma &{} \beta _0 \bar{S}&{} \kappa \beta _0 \bar{S}&{} \omega \beta _0 \bar{S}&{} \rho \beta _0 \bar{S}&{} 0 &{}0 &{} 0 &{}0 \\ \alpha \sigma &{} -r_1 &{} \nu &{} 0 &{} 0 &{} 0 &{}0 &{} 0 &{}0 \\ \left( 1- \alpha \right) \sigma &{} 0 &{} -r_3 &{} 0 &{} 0 &{} 0 &{}0 &{} 0 &{}0 \\ 0 &{} 0 &{} \varepsilon _0 &{} -r_4 &{} 0 &{} 0 &{}0 &{} 0 &{}0 \\ 0 &{} \theta &{} 0 &{} \varphi &{} -r_2 &{} 0 &{}0 &{} 0 &{}0 \\ 0 &{} \lambda &{} 0 &{} 0 &{} \tau &{} -r_5 &{}0 &{} 0 &{}0 \\ 0 &{} 0 &{} 0 &{} 0 &{} 0 &{} \delta &{}0 &{} 0 &{}0 \\ 0 &{} \eta &{} \gamma &{} \mu &{} \psi &{} \zeta &{}0 &{} 0 &{}0 \\ 0 &{} -\beta _0 \bar{S}&{} -\kappa \beta _0 \bar{S}&{} -\omega \beta _0 \bar{S}&{} -\rho \beta _0 \bar{S}&{} 0 &{}0 &{} 0 &{}0 \end{pmatrix} \end{aligned}$$

Where $r_1 = \left( \eta + \theta + \lambda \right)$, $r_2 = \left( \tau + \psi \right)$, $r_3 = \left( \varepsilon _0 + \nu + \gamma \right)$, $r_4 = \left( \varphi + \mu \right)$. and $r_5=\left( \delta + \zeta \right)$. Using $\det (sI-M) = 0$, we have the characteristic polynomial of *M* having following form: $s^{3} \left( r_{5} + s\right) p(s)$. Hence, the matrix has three null eigenvalues, one eigenvalue of $-\left( \delta + \zeta \right)$, and five eigenvalues that are roots of the polynomial *p*(*s*), where,

$$\begin{aligned} p(s) = D(s) - \bar{S}N(s) \end{aligned}$$

with,

$$\begin{aligned} \begin{aligned} D(s) = \\&s^{5} + s^{4} \left( r_{1} + r_{2} + r_{3} + r_{4} + \sigma \right) + \\&s^{3} \left( r_{1} r_{2} + r_{1} r_{3} + r_{1} r_{4} + r_{1} \sigma + r_{2} r_{3} + r_{2} r_{4} + r_{2} \sigma + r_{3} r_{4} + r_{3} \sigma + r_{4} \sigma \right) + \\&s^{2} (r_{1} r_{2} r_{3} + r_{1} r_{2} r_{4} + r_{1} r_{2} \sigma + r_{1} r_{3} r_{4} + r_{1} r_{3} \sigma + r_{1} r_{4} \sigma + r_{2} r_{3} r_{4} +\\&r_{2} r_{3} \sigma + r_{2} r_{4} \sigma + r_{3} r_{4} \sigma ) + \\&s \left( r_{1} r_{2} r_{3} r_{4} + r_{1} r_{2} r_{3} \sigma + r_{1} r_{2} r_{4} \sigma + r_{1} r_{3} r_{4} \sigma + r_{2} r_{3} r_{4} \sigma \right) +\\&r_{1} r_{2} r_{3} r_{4} \sigma \end{aligned} \end{aligned}$$

and,

$$\begin{aligned} \begin{aligned} N(s) = \\&s^{3} \beta _0 \sigma \left( 1- \alpha \right) \kappa + s^{3} \beta _0 \sigma \alpha \\&s^{2} \beta _0 \sigma \left( 1-\alpha \right) \left( \varepsilon _0 \omega + \nu + \kappa \left( r_1+r_2+r_4\right) \right) + s^{2} \beta _0 \sigma \alpha \left( \rho \theta + \left( r_2+r_3+r_4\right) \right) + \\&s \beta _0 \sigma (1-\alpha ) (\varepsilon ( \omega r_1+ \omega r_2 + \rho \varphi ) + \kappa (r_1r_2+r_1r_4+r_2r_4) + \nu (r_2+r_4+\rho \theta )) + \\&s \beta _0 \sigma \alpha (r_2r_3+r_2r_4+r_3r_4+r_3 \rho \theta + r_4 \rho \theta ) + \sigma r_1 r_2 r_3 r_4 R_0 \end{aligned} \end{aligned}$$

Defining $G(s) = N(s)/D(s)$, and noting that the system is positive, and hence $H_{\infty }$ norm of *G*(*s*) is equal to the static gain $G(0) = N(0)/D(0) = R_0$. To have the real part of every root of the polynomial to be zero or negative (Hurwitz), we should have $\bar{S}^* = {1}/{G(0)} = {1}/{R_0}$. Note that the stability of the equilibrium occurs for $\bar{S}R_0 < 1$. Since in the beginning of an epidemic we have $\bar{S}$ very close to 1, it should be noted that $R_0 < 1$ is required to have small effect and stability. $\square$

#### Fitting of the model for the COVID-19 outbreak: Italy, India and Victoria

We consider three scenarios to fit and estimate parameters for our model. First, for Italy, where the epidemic was controlled and currently have a resurgence with increasing number of cases. Second, for India, which is observing significant number of cases in recent times. Third, for Victoria, Australia, where a second re-emergence is controlled with strict intervention.

For Italy, we use data from the official source (the Department of Civil Protection—Presidency of the Council of Ministers) about the evolution of the epidemic in Italy from 2020-02-24 to 2022-03-04 (first 740 days). We convert this data to fraction of population by taking total population data from The World Bank Group (about 60297396, in 2019). The estimated parameter values are based on the data about the number of currently infected individuals that can be observed and roughly corresponding to $\left( Q(t) + H(t) + C(t)\right)$, and the number of recovered individuals that can be observed and roughly corresponding to $\int _{0}^{t}{\left( \mu Q(s) + \psi H(s) + \zeta C(s) \right) ds}$. To avoid the pitfalls described by authors in^42^, we do not fit the model to cumulative number of cases or cumulative number of deaths—however, we present them for comparison.

**Figure 12:**
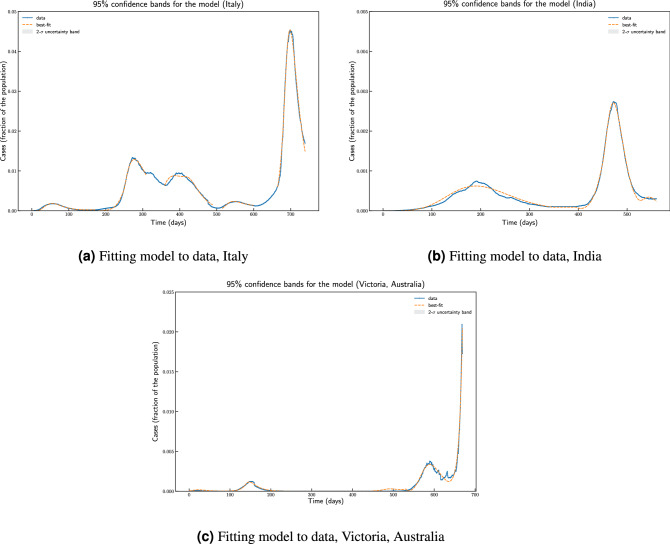
Parameter estimation and fitting model to actual data for Italy and India using lmfit. The plot show actual data, best fit by minimizing sum of the squares of the errors, and 2-$\sigma$ uncertainty band.

The ordinary differential equation (ODE) system was solved using LSODA^43,44^. We use lmfit python package^45^ for non-linear least-squares and minimize of the sum of the squares of the errors using trust region reflective method and obtaining goodness of fit measure of $\chi ^2= 5.3331e^{-07}$ for Italy. Confidence interval for the fit is shown in Fig. 12a. The problem to minimize error is shown in following equation for fitting parameter set *p*:

19 $$\begin{aligned} \hat{p} \in {\mathop {{{\,\mathrm{arg\,min}\,}}}\limits _{p}} S(p) = {\mathop {{{\,\mathrm{arg\,min}\,}}}\limits _{p}} \sum _{i=1}^{m}{\left[ y_i - f_i(x_i,p)\right] ^2 } \end{aligned}$$

where $y_i$ are observations and $f_i$ is the model output.

For India (population 1366417754, in 2019), we use data from Johns Hopkins University Center for Systems Science and Engineering (JHU CSSE). We use data from 2020-03-09 to 2021-08-04. The estimated parameter values are based on the data about the number of currently infected individuals that can be observed and roughly corresponding to $\left( Q(t) + H(t) + C(t)\right)$. In this case we obtain a goodness of fit measure of $\chi ^2= 4.4811e^{-08}$ and confidence interval for the fit is shown in Fig. 12b. Parameters $\beta _0, \beta _{\min }, \beta _{new}, t_0, t_1, r, u, \varepsilon _0, \varepsilon _{\max }, t_2$ and *s* have all been estimated from fitting the model to data. It is important to note that contact rat $\beta (t)$ and testing rate $\varepsilon (t)$ has been modelled as time variant functions.

For the state of Victoria, Australia (population 6629870, in 2019), we use data from Covid19data.com.au, which is independent and voluntarily-run. We use data from 2020-03-11 to 2022-01-13. From January 2022 onwards recovery data is not available and hence we could not compare the model with the actual number of currently active cases to which we fit our model for parameter estimation. The estimated parameter values are based on the data about the number of currently infected individuals, and we obtain a goodness of fit measure of $\chi ^2= 1.5728e^{-06}$ and confidence interval for the fit is shown in Fig. 12c.

#### Sensitivity analysis

**Figure 13:**
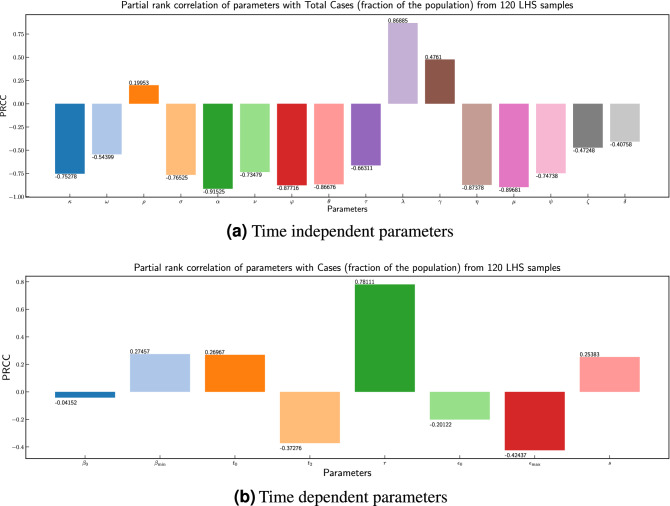
Partial rank correlation coefficients of parameters with cases (fraction of the population) from 120 LHS samples.

In this section we present the sensitivity analysis for the base model using Latin Hypercube Sampling (LHS) and Partial Rank Correlation Coefficient (PRCC) to determine the most influential parameters of the model^46^. LHS, which is a stratified sampling method, is implemented by dividing the range of values for a given parameter (see Table 1) into equally probable intervals and each parameter is sampled in the range independently. This results in a matrix having *N* rows for the number of samples and *M* columns corresponding to the number of varied parameters. We consider the increasing number of infected individuals as our outcome of interest. Base model is then simulated with population size of $1e^7$, with each row of the above mentioned metric with parameter values while setting initial values in each compartment close to 0 as per Proposition-1. Since our model has two type of parameters—one that are dependent on the time e.g. $\beta (t)$ and $\varepsilon (t)$, and others that are independent of time e.g. $\alpha , \theta$. We have performed sensitivity analysis of these parameters in two different simulations to understand their importance. The result of sensitivity analysis is given in Fig. 13. It can be seen from Fig. 13a that parameter $\alpha$ ($-0.91525$), which determines the fraction of infections that become symptomatic has most significant influence. In other words, since it has negative PRCC it implies that number of undetected asymptomatic cases can drive the cases significantly. Among the other parameters $\mu$ ($-0.89681$) and parameters related to recovery rate ($\eta , \psi$) are also influential. Among the remaining parameters rate of hospitalization also has impact.

Among the time dependent parameters $\beta (t)$, and $\varepsilon (t)$ we can observe in Fig. 13b that parameter *r*, which determines how fast $\beta (t)$ converges from $\beta _0$ to $\beta _{min}$, or effectiveness of stringency measures has significant influence. Also $\varepsilon _{\max }$, which determines the effectiveness of testing, has moderate importance.

## Discussion

In this section we consider various extension of the model and parameters. First extension is to model confinement—where a large proportion of susceptible population is placed under restricted movement, and thereby reducing the probability of contact. Such restriction has been modelled by introducing another compartment *L*. Individuals from *S* move to *L* at rate *u*(*t*). In many countries, confinement was spread over several weeks followed by a period of de-confinement. We model de-confinement by individuals moving from *L* to *S* at rate *p*(*t*). In specific we modify Eq. (9) as follows

20 $$\begin{aligned} \frac{dS}{dt} = p(t)L -\beta (t) \left( I + \kappa A + \omega Q + \rho H \right) \frac{S}{N} - u(t)S, \end{aligned}$$

We also add following dynamics to the baseline model

21 $$\begin{aligned} \frac{dL}{dt} = u(t)S - p(t)L, \end{aligned}$$

Thus, Eqs. (1–8), Eq. (20), and (21) along with Eq. (10), defines the confinement—de-confinement scenario. A few remarks are in place for the time dependent functions *u*(*t*) and *p*(*t*).

To consider the possibility of loosing acquired immunity over time and having a reinfection, we modify Eq. (9) and add a term $+\chi R$ and modify Eq. (8) by adding term $-\chi R$ to obtain:

22 $$\begin{aligned} \frac{dS}{dt}= & {} -\beta (t) \left( I + \kappa A + \omega Q + \rho H \right) \frac{S}{N} +\chi R, \end{aligned}$$

23 $$\begin{aligned} \frac{dR}{dt}= & {} \left( \eta I + \gamma A + \mu Q + \psi H + \zeta C \right) -\chi R, \end{aligned}$$

In this scenario, we assume that the susceptible individuals are vaccinated at rate $\xi (t)$. Initially $\xi (t)$ can be small and as more vaccines are produced at a larger scale, the waiting time to receive vaccine reduces (see Eq. (24)).

24 $$\begin{aligned} \xi (t) = {\left\{ \begin{array}{ll} \xi _0 &{} t < t_0 \\ \xi _{\max } - (\xi _{\max } - \xi _0) e^{-s \left( t-t_0\right) } &{} t > t_0 \end{array}\right. } \end{aligned}$$

We also assume a vaccine efficiency parameter, and assume that vaccine does not confer immunity to all vaccine recipients, and hence a vaccinated individuals may become infected but at a lower rate than un-vaccinated. Hence, by $(1-\phi )$ we denote vaccine efficiency. Thus, effective contact rate is multiplied by a scaling factor of $\phi : 0 \le \phi \le 1$, where $\phi = 0$ represents vaccine that offers $100\%$ protection against infection.

The vaccinated individuals will be denoted with compartment *V*. With that, the augmented model can be represented with following system of equations, where we replace Eq. (1) with (25), Eq. (9) with (27), remaining Eqs. (2)–(8) remains same and we add Eqs. (26), and ((28):

25 $$\begin{aligned} \frac{dE}{dt}= & {} \beta (t) \left( I + \kappa A + \omega Q + \rho H \right) \frac{S}{N} + \phi \beta (t) \left( I + \kappa A + \omega Q + \rho H \right) \frac{V}{N} - \sigma E, \end{aligned}$$

26 $$\begin{aligned} \frac{dV}{dt}= & {} \xi (t) S - \phi \beta (t) \left( I + \kappa A + \omega Q + \rho H \right) \frac{V}{N}, \end{aligned}$$

27 $$\begin{aligned} \frac{dS}{dt}= & {} -\beta (t) \left( I + \kappa A + \omega Q + \rho H \right) \frac{S}{N} - \xi (t) S, \end{aligned}$$

where,

28 $$\begin{aligned} N(t) = S(t) + E(t) + A(t) + I(t) + Q(t) + H(t) + R(t) + D(t) + V(t), \end{aligned}$$

is the total population.

**Figure 14:**
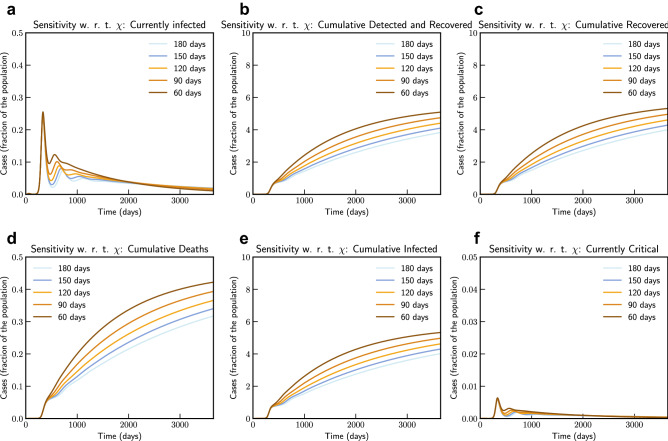
Sensitivity of $\chi (t)$ with loss of acquired immunity over time by 180, 150, 120, 90 and 60 days (Italy). Note that not all panels are in the same scale.

As described by extended model in Eqs. (22) and (23), we consider the possibility of losing acquired immunity over time and having a reinfection, we use parameter $\chi (t)$ to capture this. In our simulation we consider that $\chi$ varies from 1/180 to 1/60. This can be thought as number of days to lose immunity and hence corresponds to 180 days to 60 days. As shown in Fig. 14, the simulation of the model after fitting current data for Italy indicates that if loss of immunity occurs within 3 months, there is a significant chance of subsequent waves of epidemic.

**Figure 15:**
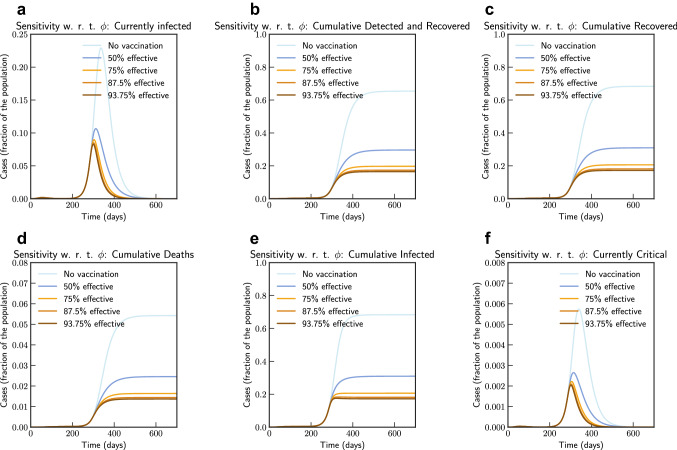
Sensitivity of vaccine efficiency parameter $(1-\phi )$, where $\phi = 0$ represents vaccine that offers $100\%$ protection against infection, and $\xi _{\max } = 0.5$, i.e. maximum of 50% population is administered with vaccine. $\phi$ is varied from 1.0 (No vaccination), 0.5 (50% efficient), 0.25 (75% efficient), 0.125(87.5% efficient), and 0.0625 (93.75% efficient) (Italy).

**Figure 16:**
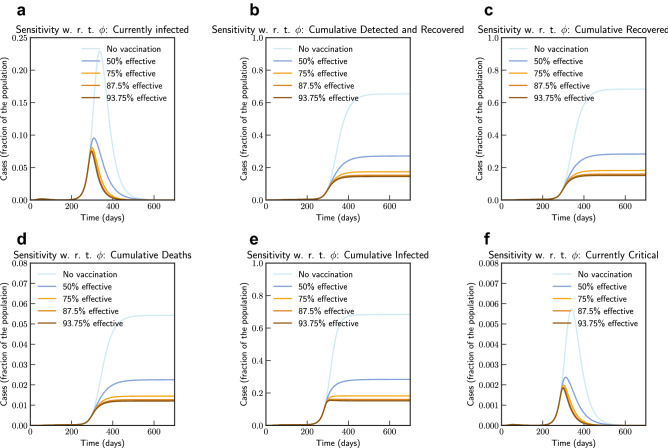
Sensitivity of vaccine efficiency parameter $(1-\phi )$, where $\phi = 0$ represents vaccine that offers $100\%$ protection against infection, and $\xi _{\max } = 0.9$. $\phi$ is varied from 1.0 (No vaccination), 0.5 (50% efficient), 0.25 (75% efficient), 0.125(87.5% efficient), and 0.0625 (93.75% efficient) (Italy).

Finally, we simulate mass vaccination scenarios (see Figs. 15 and 16) according to Eqs. (25), (26), (27), (28) and (24). We assume that vaccine is available after 90 days from 27th August 2020. $\phi$ is varied from 1.0 (No vaccination), 0.5 (50% efficient), 0.25 (75% efficient), 0.125(87.5% efficient), and 0.0625 (93.75% efficient) and $\xi _{\max } = 0.5$ and $\xi _{\max } = 0.9$. It can be concluded that even a 75% efficient vaccine can be significantly effective in reducing the impact.

**Figure 17:**
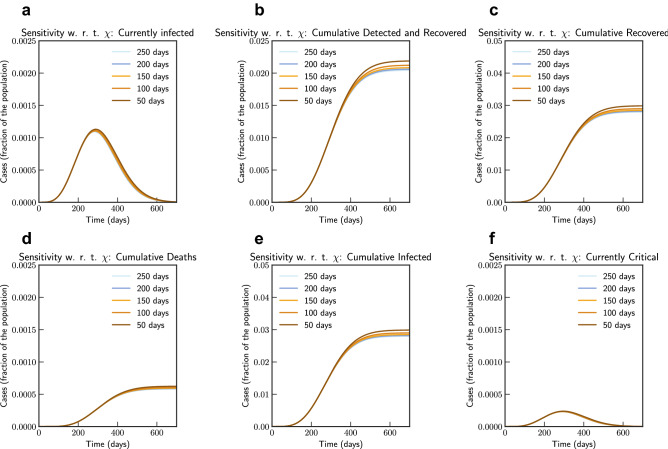
Sensitivity of $\chi (t)$ with loss of acquired immunity over time by 250, 200, 150, 100 and 50 days (India). Note that not all panels are in the same scale.

Figure 17 show the simulation study loss of acquired immunity for scenario in India. We note that this model is not very sensitive to $\chi (t)$. This is due to the size of the population and current state of the epidemic in India—reinfection within 2 months changes total number of infected population from 2.66 to 2.98%, while reinfection after 250 days changes this to 2.768%.

**Figure 18:**
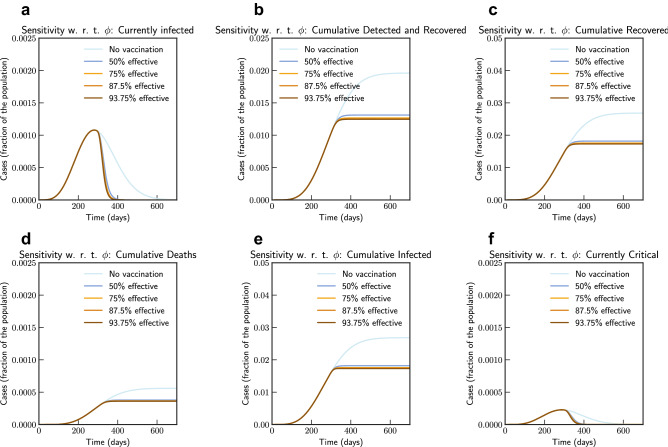
Sensitivity of vaccine efficiency parameter $(1-\phi )$, where $\phi = 0$ represents vaccine that offers $100\%$ protection against infection, and $\xi _{\max } = 0.3$, i.e. maximum of 30% population is administered with vaccine. $\phi$ is varied from 1.0 (No vaccination), 0.5 (50% efficient), 0.25 (75% efficient), 0.125(87.5% efficient), and 0.0625 (93.75% efficient) (India).

**Figure 19:**
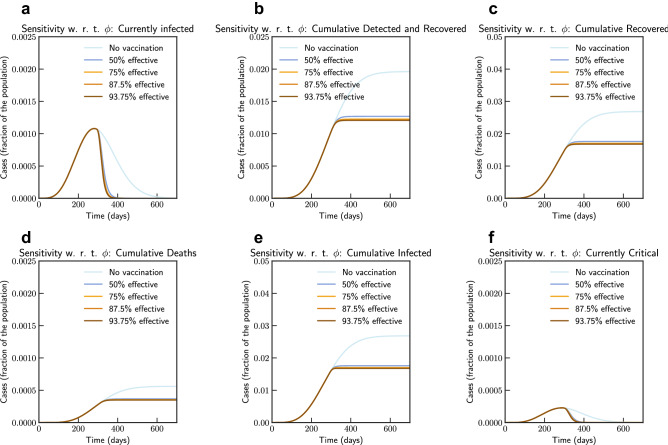
Sensitivity of vaccine efficiency parameter $(1-\phi )$, where $\phi = 0$ represents vaccine that offers $100\%$ protection against infection, and $\xi _{\max } = 0.7$, i.e. maximum of 70% population is administered with vaccine. $\phi$ is varied from 1.0 (No vaccination), 0.5 (50% efficient), 0.25 (75% efficient), 0.125(87.5% efficient), and 0.0625 (93.75% efficient) (India).

Simulation studies for vaccination is presented in Figs. 18 and 19. We assume that the vaccine is available from 90 days after 16th September 2020. Simulation suggests that a 0.056% of population would die without vaccination, while 93.75% efficient vaccine given to 30% population would bring this down to 0.036% of population, and 93.75% efficient vaccine given to 70% population would bring this down to 0.034%.

## Conclusion

In this work we have presented a deterministic compartmental model that is an extension of the SEIR model in which we have included current experience with SARS-CoV-2—namely, we have extended standard SEIR model to include symptomatic and asymptomatic classes, and the fact that in SARS-CoV-2, as it has been observed that asymptomatic classes can infect susceptible individuals. We have then extended this base model to address various scenarios including the effects of non-pharmaceutical interventions (such as social distancing, self-isolation, contact tracing etc.) and pharmaceutical interventions (such as testing, mass vaccination with variable efficacy and variable percent of population being vaccinated). Scenario also includes the possibility of losing acquired immunity over time and reinfection.

In particular, our simulation of the model in various scenarios (Figs. 14, 15, 16, 17, 18, 19) suggests that shorter reinfection time, and/or usage of a vaccine of lower efficacy fails to control the exponential growth of infection in populations which is highly susceptible to the disease (e.g. because they are far from reaching herd immunity), and induces wave like growth patterns (with re-infection). In practice, such scenario indicates that continued surveillance and re-introduction of non-pharmaceutical interventions remains necessary for longer term.

In order to estimate various parameters we have used published data in three different scenarios—in Italy, where in initial stages there were a growing number of cases and re-emergence of the epidemic, in India, where there were significant number of cases post confinement period and in Victoria, Australia where a re-emergence has been controlled with severe social confinement program. Following the initial stages we have used data to estimate various parameters during the emergence and spread of the Alpha B.1.1.7, Delta B.1.617.2, and Omicron B.1.1.529 VOCs. The estimated parameter values are based on the data about the number of currently infected individuals that can be observed and roughly corresponding to $\left( Q(t) + H(t) + C(t)\right)$, and the number of recovered individuals that can be observed and roughly corresponding to $\int _{0}^{t}{\left( \mu Q(s) + \psi H(s) + \zeta C(s) \right) ds}$. We obtain a goodness of fit measure between $\chi ^2=[157.28-4.4811]e^{-08}$ across these data sets. Sensitivity analysis of parameters of our model implies that number of undetected asymptomatic cases can drive the growth significantly and which in practice implies the importance of contact tracing and testing. This is corroborated further by importance of parameters that relate to effectiveness of stringency measures—which has significant influence. Also the fact that parameter which determines the effectiveness of testing, has moderate importance.

Our result shows the benefit of long term confinement of at least half of population and extensive testing in curbing exponential growth. With respect to loss of acquired immunity, our model suggests higher impact for a country with population like Italy. We also show that a reasonably effective vaccine with mass vaccination program are successful measures in significantly controlling the size of infected population. We show that for a country like India, a reduction in contact rate by half could reduce potential death by half in initial stages of the epidemic. Similarly, for a country like Italy, we show that reducing contact rate by half can reduce a potential peak infection and potential deaths by ten fold. With respect to vaccination, we show that even a 75% efficient vaccine administered to 50% population can reduce the peak number of infected population by nearly 50% in Italy. Similarly, for India, a 93.75% efficient vaccine given to 70% population would reduce potential death by half. These results might be useful in continued global effort in curbing exponential growth from ongoing and emerging future VOCs.

## Data Availability

All of the data are publicly available and were extracted from https://github.com/M3IT/COVID-19_Data, from https://github.com/pcm-dpc/COVID-19, and from https://github.com/CSSEGISandData/COVID-19.

## Code availibility

Python codes were used for model implementation. All source codes can be accessed from https://github.com/subhaskghosh/model_covid_19.
